# Surgical treatment for fibrous dysplasia of femoral neck with mild but prolonged symptoms: a case series

**DOI:** 10.1186/s13018-015-0208-6

**Published:** 2015-05-10

**Authors:** Yoshihiro Nishida, Satoshi Tsukushi, Kozo Hosono, Hiroatsu Nakashima, Yoshihisa Yamada, Hiroshi Urakawa, Naoki Ishiguro

**Affiliations:** Department of Orthopaedic Surgery, Nagoya University Graduate School and School of Medicine, 65 Tsurumai, Showa, Nagoya, Aichi 466-8550 Japan; Orthopaedic Surgery, Aichi Cancer Center Aichi Hospital, 18 Kuriyado, Kake-machi, Okazaki City, Aichi Japan; Orthopaedic Surgery, Nagoya Memorial Hospital, 4-305 Hirabari, Tenpaku-ku, Nagoya, Japan; Present address: Orthopaedic Surgery, Gifu Prefectural Tajimi Hospital, 5-161, Maehata, Tajimi, Gifu Japan

**Keywords:** Fibrous dysplasia, Femoral neck, Fibula strut, β-TCP, Bone regeneration

## Abstract

**Background:**

The proximal femur is one of the most common sites involved by fibrous dysplasia. In cases with mild deformity that does not require corrective surgery, occasional patients suffer sustained pain because of repeated microfractures. This study aimed to clarify the outcomes of surgery with autogenous fibular cortical strut grafting and compression hip screw fixation.

**Methods:**

Since 2002, eight consecutive patients (nine hips) with femoral neck fibrous dysplasia without severe deformity were prospectively treated with autogenous fibular strut grafting and compression hip screw fixation.

**Results:**

Mean age of patients was 35 years. Mean follow-up of patients after surgery was 75 months. Most of the patients could walk with full weight-bearing 2 weeks after surgery. Functional score of lower extremity was significantly improved from 65 % to 95 % (*P* = 0.001). Femoral neck angle was increased from 127 to 130. Donor site of strut cortical fibula showed good regeneration with β-tricalcium phosphate.

**Conclusions:**

Autogenous fibular cortical strut grafting and compression hip screw fixation achieved good post-operative function and provided an early return to work for adult patients with fibrous dysplasia of the femoral neck with mild but prolonged symptoms. Morbidity in the donor site of fibula strut is minimal with the use of β-tricalcium phosphate.

## Introduction

Fibrous dysplasia is a benign fibro-osseous lesion first reported by Lichtenstein and Jaffe [1]. It is thought to occur as a result of a developmental failure of bone. Normal lamellar bone is replaced with irregular trabeculae of woven bone intermixed with mature collagenous tissue, resulting in insufficient mineralization, leading to substantial loss of mechanical strength, and subsequently, pain, deformity, and pathologic fractures. Fibrous dysplasia may affect one bone (monostotic) or multiple bones (polyostotic). GNAS (guanine nucleotide-binding protein/α-subunit) mutations participate in the pathogenesis of fibrous dysplasia and have a diagnostic significance [2]. Although malignant transformation of fibrous dysplasia is rare, early diagnosis and adequate treatment may lead to favorable prognosis [3].

Sometimes monostotic fibrous dysplasia can cause hip pain and/or a fatigue fracture in adolescence and early adulthood [4] that impair the normal activities of daily living (ADL). Surgical intervention is required for these cases with mild, but prolonged symptoms.

The relatively early literature reported the clinical results of surgical treatment for fibrous dysplasia of the femoral neck including autogenous cancellous bone graft, autogenous cancellous bone and allograft, fibular graft, and fixation of compression hip screw (CHS) in addition to autogenous cancellous bone and allograft [4–12]. Their fundamental concept for surgical treatment is that fibrous dysplasia of the femoral neck with fatigue fractures should be treated without aggressive resection of tumors, fatigue fractures should be repaired, and additional fractures and deformity should be avoided. However, these previous reports did not assess the function of the involved extremity pre- and post-operatively.

Surgical treatment is recommended for repeated or persistent pain due to microfractures. These cases typically manifest parrot’s beak sign, which radiographically represents a thin radiolucent fracture line between bony callus on the medial cortex, resembling the appearance of the beak of a bird. Most of these patients had some difficulties in ADL but could pursue their social activities pre-operatively. Together, surgical treatment should be planned for these patients to improve the function of the extremity and to facilitate resumption of a full daily life as soon as practicable.

Since 2002, patients with fibrous dysplasia of the femoral neck, who had mild, but persistent pain, have been consecutively and prospectively treated with autogenous fibular strut grafting and fixation with CHS in our institutions. Clinical outcomes were analyzed radiographically and functionally. In addition, the fibular graft donor site, where β-tricalcium phosphate was implanted, was evaluated.

## Materials and methods

**Fig. 1:**
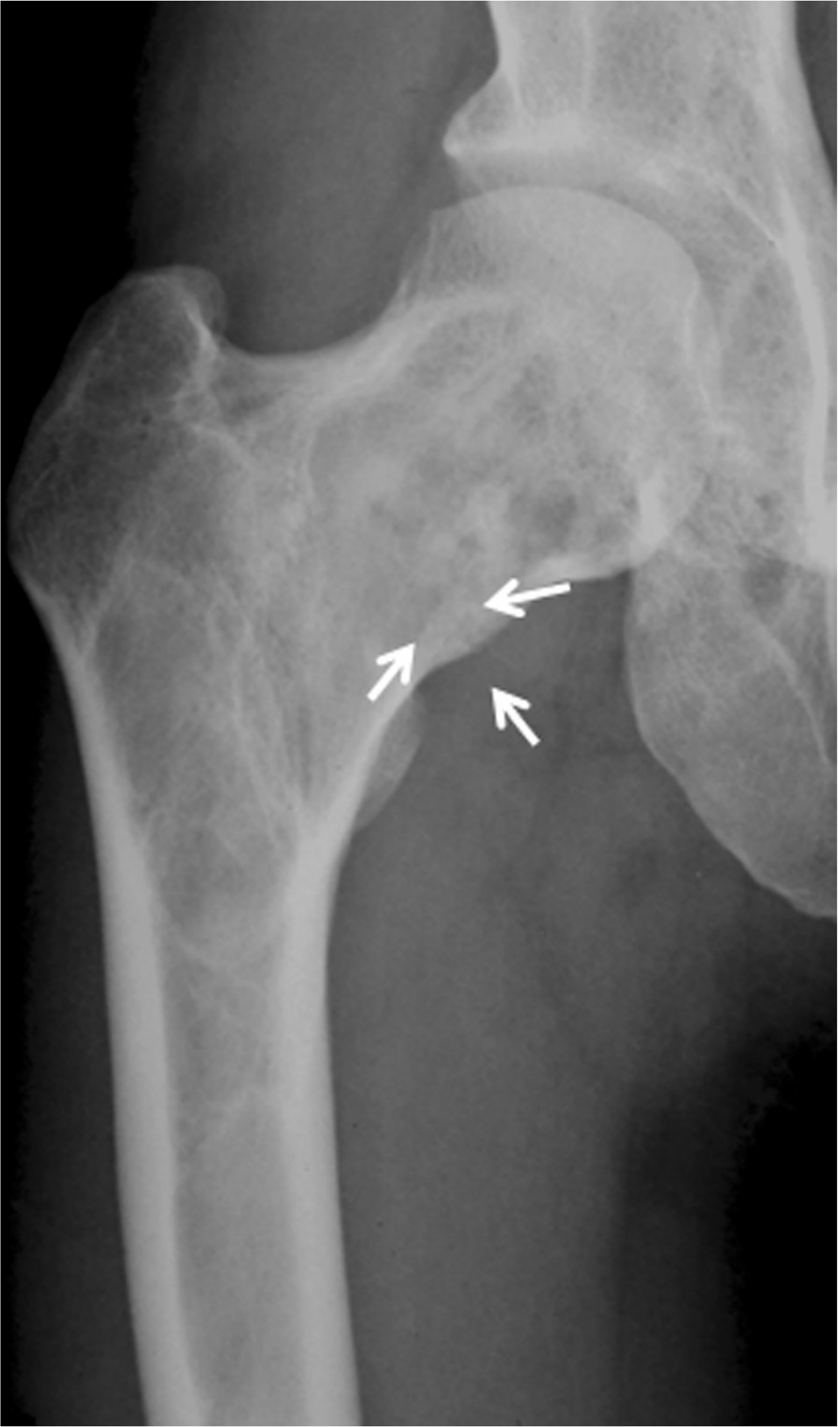
Patient 4. A 32-year-old female presented with repeated bilateral hip pain for 20 years. An A-P X-ray at the time of initial referral indicated fibrous dysplasia of her right femoral neck and “parrot’s beak” sign (*arrows*)

This study was approved by the Institutional Review Board in our institution, and the use of their medical information was permitted by the patients. From 2002 to 2012, 236 patients were radiographically diagnosed with fibrous dysplasia in our institutions (NU, ACC, NMH), 103 of whom had fibrous dysplasia in femurs. Eight patients (nine hips) had mild, but persistent pain with slight deformity of the femoral neck, mostly parrot’s beak sign on antero-posterior (A-P) X-ray view (Fig. 1). Surgical indications of this cohort included a large lesion of fibrous dysplasia in the femoral neck region associated with impending or actual fracture (evaluated as parrot’s beak sign) leading to persistent or repeated pain. Cortical fibula graft was indicated in patients who had sufficient normal bone in both the proximal (femoral head) and distal lateral cortex of the proximal part of the femur to anchor the graft at both ends in normal bone. These patients were prospectively, and consecutively treated with surgery; pathological diagnosis intraoperatively, without curettage, autogenous fibular strut graft, and fixation of CHS. There were two males (two hips) and six females (seven hips). Among the eight patients (nine hips), two had polyostotic and six monostotic involvement. None of the patients were thought to have definitive findings of McCune-Albright syndrome or Mazabrauds syndrome. Osteofibrous dysplasia was also excluded by image analysis and pathological determination. Six hips had a pathological fracture (parrot’s beak sign) pre-operatively. All patients with pathological fractures were female. Treatment modality was not changed between male and female. Mean and median ages at surgery were 35 and 32 (ranging from 22 to 69) years, respectively. Detailed information regarding patient gender, age at the time of surgery, type of fibrous dysplasia, and existence of pathological fracture is listed in Table 1.

**Table 1:** Clinical data for patients with fibrous dysplasia in femoral neck (all cases)

Case	Sex	Age	Type of FD	Pathological fracture	Length of fibula graft (cm)	FU (months)	BU (months)
1	F	22	Mono	Yes	9.0	127	5
2	F	23	Poly	Yes	8.0	96	3
3	M	32	Mono	No	10.5	45	3
4 (R)	F	32	Poly	Yes	9.0	128	4
4 (L)	F	32	Poly	Yes	9.0	128	8
5	M	69	Mono	No	9.0	36	12
6	F	41	Mono	Yes	8.8	31	7
7	F	21	Mono	No	8.4	12	4
8	F	44	Mono	Yes	8.0	74	7

Abbreviations: FD, fibrous dysplasia; FU, follow-up duration; BU, bone union; R, right side; L, left side; F, female; M, male; Mono, monostotic; Poly, polyostotic

Prior to surgical treatment, A-P and lateral plain radiographs of the proximal femur were taken to determine the neck-shaft angle of the implant and to evaluate the ipsilateral side of the fibula as a donor site for the strut cortical bone graft. A histological diagnosis was not made prior to the definitive surgery because the roentogenographic findings were typical of fibrous dysplasia of the femoral neck region.

**Fig. 2:**
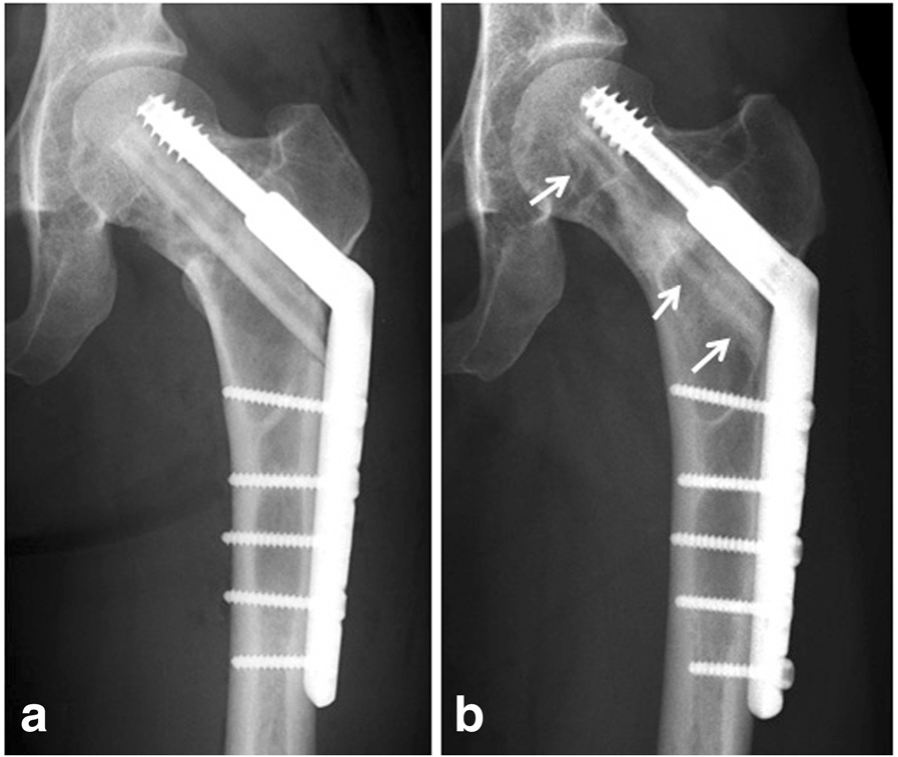
Patient 4. X-ray at the time of surgery after fixation with a compression hip screw (**a**). Nine years after surgery, a grafted fibular strut was well preserved (**b**, *arrows*)

The surgical procedure used in the present cohort is as follows. The patient is positioned on a fracture table, allowing the C-arm fluoro-image intensifier to be positioned between the patient’s legs. A lateral straight incision is made over the fibula ipsilateral to the femoral neck fibrous dysplasia to obtain the cortical fibular autograft. The length of the fibula required for the graft was calculated by A-P views; from lateral cortex of femoral shaft to 1 cm below the hip joint space along the calcar femorale. The anticipated length of the fibula is harvested using an oscillating saw. Periosteum of the fibula is carefully preserved, and β-tricalcium phosphate (β-TCP) is used as a bone graft substitute to backfill the fibular defect [13]. Periosteum is sutured to cover the implanted β-TCP. A lateral straight incision is made to the proximal femur from the greater trochanter extending distally along the femoral shaft. An appropriate fixed-angle plate (125°–135°) is placed on the lateral cortex, so that the guide pin can be aimed toward the normal bone in the femoral head. Preferably, the guide pin is inserted parallel and adjacent to the calcar femorale. The position of the guide pin is evaluated and confirmed on both A-P and lateral views. The femur is reamed with a cannulated reamer to create a tunnel for insertion of the fibular strut graft. At the beginning of the reaming, tissues of the tumorous lesion are obtained and subjected to examination of the frozen section. In all cases, histological examination confirmed the diagnosis of fibrous dysplasia. Basically, no additional curettage is performed. The diameter of the reamer is slightly less than that of the graft. Next, fibular strut bone is inserted with a hammer. Generally, tissues of fibrous dysplasia are moderately rigid, making it necessary for the operator to use a hammer to insert the graft. The grafted fibular strut is positioned from the lateral aspect of the cortex to the normal bone of the femoral head. No additional bone grafts are used other than the fibular strut graft. Subsequently, the femoral neck is fixed with CHS. A guide wire is placed in the femoral neck superior and parallel to the grafted fibular strut. A lag screw is inserted juxtaposed to the grafted fibular strut and then a side plate is attached to the lag screw. Bone screws are inserted with a conventional method (Fig. 2). Mean duration of operation time and blood loss during operation was 246 minutes and 372 ml, respectively. Patient 4 was a polyostotic type and the fibrous dysplasia of both femoral necks was operated on at the same time. Mean length of the strut fibula graft was 9 cm (range, 8.0–10.5) (Table 1).

Post-operatively, weight-bearing is allowed after patients recover from the surgery. Time of initiation for full weight-bearing walking is determined according to the patient’s symptoms, rather than the radiographic findings. Patients undergo follow-up examinations at the outpatient unit every three months for the first year and every six months thereafter. Mean follow-up after surgical treatment was 75 months (range, 12–128) (median, 74 months).

The clinical results are evaluated with MSTS (Musculoskeletal Tumor Society)-ISOLS (International Society of Limb Salvage) system [14]. These criteria include numerical values (0–5) for each of six categories: pain, function, emotional acceptance, supports, walking, and gait in the lower extremity. We evaluated the pre- or post-operative function using five categories excluding emotional acceptance. At the examination of each follow-up, neck-shaft angle of the femur, incorporation and/or absorption of the grafted strut fibula, size of the fibrous dysplasia in the femoral neck, and regeneration of the donor site of fibula, where β -TCP was implanted, are evaluated.

### Statistical analyses

We determined differences in MSTS-ISOLS functional score between the pre-operative and post-operative status using the two-tailed student *t*-test. All analyses were performed using SPSS 17.0 for Windows software. *P* < 0.05 was considered statistically significant.

## Results

**Fig. 3:**
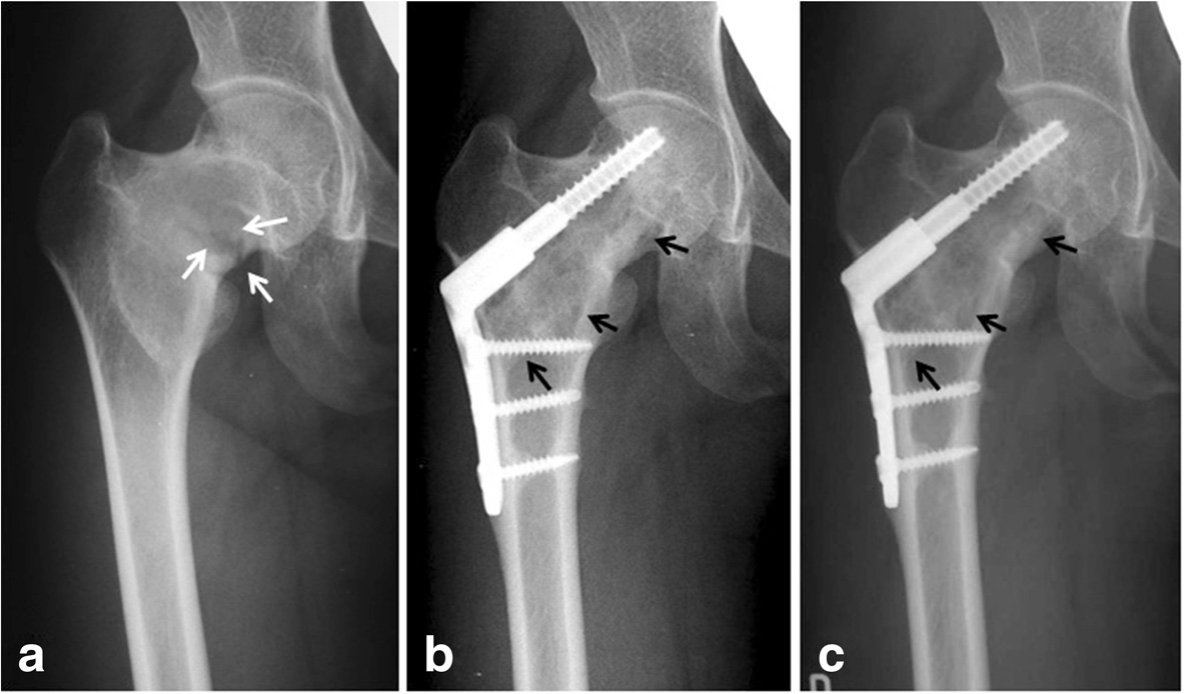
Patient 1. A 22-year-old female presented with repeated or persistent right hip pain since for ten years. An A-P X-ray at the initial presentation indicated fatigue fracture with “parrot’s beak” sign (*white arrows*) (**a**). X-rays at three years (**b**) and ten years (**c**) after surgery revealed cure of the fatigue fracture and preservation of the grafted autogenous fibular strut (*black arrows*)

In six hips with fatigue fracture, the “parrot beak” lesion seen radiographically was cured after fibular grafting and CHS surgery. Up to the final follow-up, no fatigue fractures recurred in the femoral neck region. The mean neck-shaft angle of the affected femur was 127° (range, 114°–140°) and that of the unaffected femur was 135° (127°–145°) pre-operatively. The post-operative angle of the affected femur was improved to 130° (mean range, 118°–140°) (Table 2), probably due to the implanted fixed-angle plate (125°–135°). Plain radiographs showed that the size of the lesion was unchanged in six hips, and slightly decreased in three hips, consistent with the findings in a previous report [4]. Partial or complete structural continuity of the grafted fibular strut was confirmed in all nine hips with plain radiographs at the last follow-up (Figs. 2 and 3). Bone union was observed between grafted fibula and host bone at six months after surgery (mean ranging from 3–12 months).

**Table 2:** Pre- and post-operative status of all patients

Case	Neck-shaft angle (°) (pre-)	Neck-shaft angle (°) (post-)	Functional score (pre-)	Functional score (post-)
1	115	118	20	25
2	140	140	17	25
3	136	139	21	25
4 (R)	126	127	16	25
4 (L)	127	130	16	25
5	118	120	23	23
6	133	133	6	21
7	132	135	10	24
8	114	130	18	25

Functional score, MSTS-ISOLS functional score; full score is 25 points.

Abbreviations: pre-, pre-operative; post-, post-operative

**Fig. 4:**
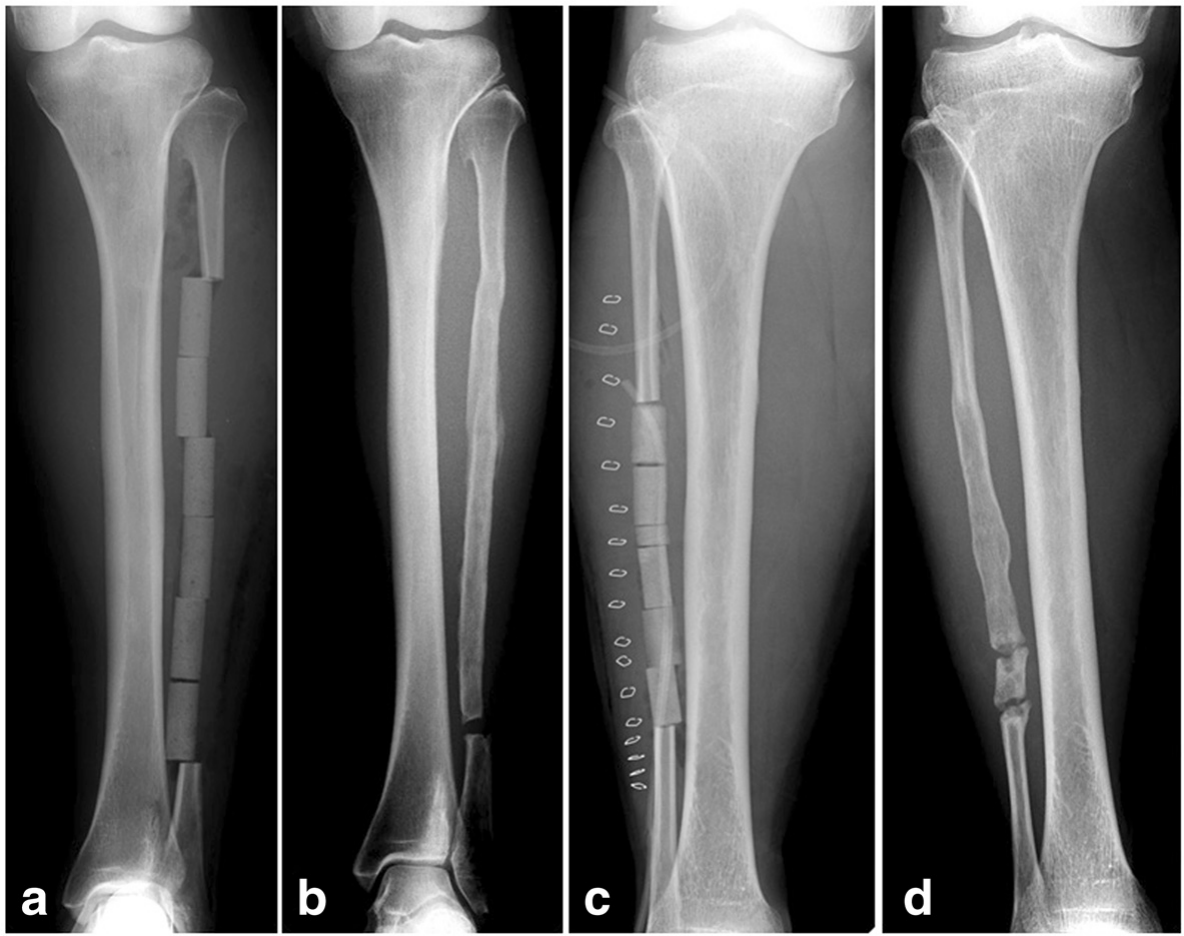
Donor sites of fibular strut. (**a**) Patient 4. β -TCPs were implanted at the donor site of fibula. (**b**) Eight years after surgery, the implanted β -TCPs were replaced by newly formed bone. (**c**) Patient 5, a 69-year-old male. A-P X-rays soon after surgery (**c**) and three years after surgery (**d**) indicated regenerated new bone formation

β-TCP in the donor site was replaced to various degrees by newly formed bone in all patients [13]. In seven patients, more than 80 % of the length was regenerated with newly formed bone, although all nine hips had nonunion at the distal junction of β-TCP with residual host distal fibula (Fig. 4).

Five of eight patients (six of nine hips) could walk with full weight-bearing by two weeks after operation. All patients became asymptomatic within two months after surgery. The mean MSTS-ISOLS functional score was 65 % (range, 24–92 %) pre-operatively, and 96 % (range, 84–100 %) post-operatively (Table 2). Scoring was significantly improved post-operatively (*P* = 0.001). No significant complications were observed in any patients at the donor site for the graft or at the femoral neck region. No implant of CHS was removed during the follow-up in any patient, and no implant-related complications were observed.

## Discussion

The present study reported patients with fibrous dysplasia of the femoral neck, prospectively and consecutively treated with identical methods in all nine hips, namely autogenous fibular strut grafting combined with CHS. Several factors need to be considered when deciding on the surgical procedure for fibrous dysplasia, particularly of the femoral neck. Most of the cases in the present study were of the mildest type of deformity according to the patterns recently reported [15]. Another factor is the patient’s age. Monostotic lesions remain active only until skeletal maturity. Given that all patients in the present study were over 20 years at the time of surgery, where six had monostotic involvement, invasive surgical procedures should be avoided, and procedures allowing patients to return to their usual activities as early as possible are desired.

Several previous studies reported radiographic evaluation after the treatment of fibrous dysplasia in the femoral neck with various types of grafts (Table 3). Nakashima *et al*. [8] reported eight cases with monostotic disease of the femoral neck treated with curettage and autogenous bone-grafting. The lesion resolved in six cases, although they did not describe the type of graft used. Harris *et al*. [7] reported on ten patients with fibrous dysplasia of the femoral neck treated with curettage and autogenous bone graft. Five had a poor result. Guille *et al*. reported on a larger series of lesions treated with curettage and autogenous cancellous bone-grafting [6]. In their study, at the time of the last follow-up, complete resorption of all autogenous cancellous bone grafts was observed radiographically. Taking these previous observations together, autogenous cancellous bone can be assumed to be replaced by host bone, and so physicians have tended to use cortical bone grafts.

**Table 3:** Previous reports describing the surgical treatment for patients with fibrous dysplasia of proximal neck

	No. of cases	Type of bone graft	Internal fixation	Clinical course
Nakashima *et al*. [8]	8	Autograft	None	six of eight patients were successful
Enneking *et al*. [3]	15	Cortical autograft	None	Continuity of grafted bone in all cases, two cases required additional surgery
Harris *et al*. [7]	10	Autograft	None	five had a poor result
Shih *et al*. [12]	22	Cortical allograft, cancellous autograft	CHS	All patients had good bone healing
Guille *et al*. [6]	22	Cancellous autograft	Yes*	All cancellous bone grafts were resorbed
Current study	8	Cortical autograft	CHS	All patients had improved post-operative function

*methods varied, CHS; compression hip screw

Enneking *et al*. [4] reported 15 relatively undeformed cases with cortical fibular autografts without internal fixation, which might be categorized as the mildest form of deformity in the femoral neck according to the classification of Ippolito *et al*. [15]. All cases showed continuity and integrity of the grafts on the final follow-up radiographs. However, slow resorption of the initial graft was observed associated with the development of pain and fatigue fracture in two cases. Intriguingly, both of these cases were monostotic. In the present study, no cases had recurrence of fatigue fractures or recurrent pain.

Springfield proposed the possible superiority of cortical bone allografts because of their availability and a decreased rate of resorption compared with autogenous cortical bone grafts [16]. Other authors outlined the advantage of allografts due to their unlimited supply and absence of additional donor site morbidity [17]. The process of the incorporation of allografts is slower and probably less complete than those associated with autografts [18, 19], while the present study revealed that all autogenous fibular strut grafts remained at the grafted sites during the mean follow-up of 75 months. Several disadvantages of the acquisition of autogenous fibular cortical bone have been reported including weakened donor site, higher post-operative morbidity, the need for a second surgical incision to obtain donor fibula, and limited availability of donor bone [20]. However, grafted β-TCP was well replaced by autogenous regenerated bone in all of the present cases as previously reported [13]. Preservation of periosteum is essential for the regeneration of bone, and grafted β-TCP is completely replaced by host bone, which might be different from the graft of hydroxyapatite. No significant morbidity was observed, and availability of the fibula was not problematic for the present patient cohort with only a minimally deformed femoral neck.

One of the purposes in the present study was to evaluate post-operative function. Previous reports described the functional course after surgical treatment. In the Enneking *et al*. [4] study, their patient used crutches for six weeks post-operatively, and partial weight-bearing was allowed thereafter. Considering that the mean age of their cohort was 20 years, social activities should have been allowed as soon as practicable. Shih *et al*. reported femoral neck cases of fibrous dysplasia treated with curettage, cancellous bone grafts (auto- or allografts), cortical strut allograft, and fixation of compression hip screw [10, 12]. In their report, a large cortical window was required to achieve complete curettage. However, curettage of the tumor would not be necessary in cases with the mildest form. The patients in their analyses were allowed to ambulate with full weight-bearing after 6 weeks, although to us this seemed to be an excessively long duration given that their patients were young with a mean age of 20 years with only mildly deformed femoral necks. In our cohort, most of the patients could walk with full weight-bearing by two weeks after surgery, which is considered to be adequate because the patients received minimum curettage and CHS augmentation. According to the MSTS-ISOLS functional rating score, the results were good or excellent in their (Shih et al.) studies. However, there was no comparison with the pre-operative status, and the actual score was not noted. The present study, in which the MSTS-ISOLS functional score was evaluated in all cases both pre- and post-operatively, demonstrated that scores were significantly improved post-operatively compared with those evaluated pre-operatively. Another case report described the use of a fibular strut graft for fibrous dysplasia of the femoral neck [5], in which, however, there were no signs of fatigue fracture on plain X-ray, although the follow-up period was only one year, and no functional evaluation was achieved.

This study had several limitations. Only a small number of cases could be studied because we focused on the mildest deformed form of fibrous dysplasia in the femoral neck [15]. However, this case series was prospectively and consecutively treated with identical surgical procedures, and the mean follow-up duration was relatively long compared with those noted in previous reports, adding to the validity of the data. CHSs were not removed in any cases during follow-up because the patients had no problems with the devices in the pursuance of their normal daily and sports activities. The low availability of bone allografts in our country may encourage physicians to use autogenous fibular struts, the disadvantages of which are partly overcome by using β-TCP after acquisition of fibular struts.

## Conclusions

Autogenous fibular cortical strut grafting and compression hip screw fixation achieved good post-operative function and early return to work for adult patients with fibrous dysplasia of the femoral neck with mild but prolonged symptoms. Compared with cortical allografts, although there is some morbidity at the fibula strut donor site, it will be mitigated by use of β-TCP.

## Abbreviations

ADL: Activities of daily living
CHS: Compression hip screw
A-P: Antero-posterior
β-TCP: β-tricalcium phosphate
MSTS: Musculoskeletal tumor society
ISOLS: International Society of limb salvage
